# Genomic and functional characterization of carbapenem-resistant *Klebsiella pneumoniae* from hospital wastewater

**DOI:** 10.1186/s12866-023-02862-5

**Published:** 2023-04-24

**Authors:** Zhiqiang Xie, Jiangqing Huang, Shengcen zhang, BinBin Xu, Qianwen Zhang, Bin Li

**Affiliations:** Department of Clinical Laboratory, Fujian Medical University Union Hospital, 29 Xinquan Rd, Fuzhou, 350001 Fujian China

**Keywords:** Carbapenem-resistant *Klebsiella pneumoniae*, Hospital wastewater, Whole genome sequencing, Phylogeny, *Bla*_KPC-2_

## Abstract

**Background:**

The emergence of carbapenem-resistant *Klebsiella pneumoniae* (CRKP) attracted extensive attention. Information on CRKP from hospital wastewater (HWW) is limited. The aims of this study were to investigate the genomic characteristics and to evaluate the survivability characteristics of 11 CRKP from HWW in a Chinese teaching hospital in Fujian province.

**Results:**

A total of 11 CRKP from HWW were recovered in this study. All CRKP from HWW were resistant to most antibiotics. Comparative genetic analysis demonstrated that all CRKP isolates were clustered into the three distinct phylogenetic clades and clade 2 and clade 3 were mixtures of samples collected from both HWW and clinical settings. Varieties of resistance genes, virulence genes and plasmid replicon types were detected in CRKP from HWW. In vitro transfer of *bla*_KPC-2_ was successful for 3 *bla*_KPC-2_-positive CRKP from HWW with high conjugation frequency. Our study demonstrated that the genetic environments of *bla*_KPC−2_ shared core structure with IS*Kpn27*-*bla*_KPC−2_-IS*Kpn6*. Group analysis showed that CRKP from HWW had a lower survivability in serum compared to clinical CRKP (*p* < 005); and CRKP from HWW had no significant difference in survivability in HWW compared to clinical CRKP (*p* > 005).

**Conclusions:**

We analyzed the genomic and survivability characteristics of CRKP from HWW in a Chinese teaching hospital. These genomes represent a significant addition of genomic data from the genus and could serve as a valuable resource for future genomic studies about CRKP from HWW.

**Supplementary Information:**

The online version contains supplementary material available at 10.1186/s12866-023-02862-5.

## Background

The emergence of carbapenem-resistant *Klebsiella pneumoniae* (CRKP) attracted extensive attention, which was a significant public health challenge worldwide [1]. CRKP can cause many kinds of infections, such as pneumonia, bloodstream infections and urinary tract infections, and responsible for substantial patient morbidity and mortality [2].

Notably, many CRKP isolates were recovered from the environments, especially the water environment, such as the wastewater, rivers and lakes [3–5]. Hospital wastewater (HWW) was recognized as a putative reservoir for CRKP [6]. Current evidence concurs that HWW was an important source for antibiotic resistance in aquatic environments, mainly multidrug-resistant Gram-negative bacteria[7]. Compared to other wastewaters, antibiotic-resistant Gram-negative bacteria were more prevalent in HWW, with higher concentrations of extended spectrum beta-lactamase (ESBLs)-producing pathogens and carbapenemase-producing *Enterobacterales* in hospital sources [7].

There are substantial studies regarding the global prevalence and trends of clinical CRKP [6]. Many previous studies of HWW mainly focused on the prevalence of carbapenem-resistant genes, molecular characteristic of bacteriophage in CRKP and CRKP infection or colonization associated with intensive care unit sewage in China [3, 4, 8, 9]. Although there was increased research on CRKP from HWW epidemiology and phylogenomic worldwide [6], large gaps remain in our understanding regarding the genomic and functional characterization of CRKP from HWW, especially in China. Therefore, the aims of this study were to investigate the genomic characteristics using whole genome sequencing and to evaluate the survivability characteristics of CRKP from HWW, which could help us to obtain a comprehensive understanding of CRKP from HWW.

## Results

### Bacterial isolates and antimicrobial susceptibility testing

In this study, hospital wastewaters contain CRKP approximately at 1.39 × 10^4^ CFU/ml using serial diluted and a total of 11 CRKP isolates were recovered. All CRKP from HWW were resistant or intermediate to most antibiotics, including ertapenem (100%, MIC values ranged from 8 to 128 μg/mL), imipenem (100%, MIC values ranged from 8 to 64 μg/mL), meropenem (100%, MIC values ranged from 8 to 128 μg/mL) and tigecycline (100%, MIC values ranged from 4 to 16 μg/mL). 91.8% of CRKP isolates (from HWW) were interpreted as intermediate (≤ 2 μg/mL, MIC values ranged from 1 to 64 μg/mL) for colistin (Table 1).

**Table 1:** Antimicrobial Susceptibility Testing of CRKP from HWW and Transconjugants

Antibiotics antimicrobial susceptibility (MICs(mg/L))	W3A23	W3A63	W3A65	W3A70	W25A158	W25A96	W25A97	W3A17	W3A37	W3A87	W25A58	Transconjugant (W25A96- *bla*_*KPC*-2_)	Transconjugant (W25A97- *bla*_*KPC*-2_)	Transconjugant (W25A158- *bla*_*KPC*-2_)	EC600
Ampicillin	$\geqq$32/R	$\geqq$32/R	$\geqq$32/R	$\geqq$32/R	$\geqq$32/R	$\geqq$32/R	$\geqq$32/R	$\geqq$32/R	$\geqq$32/R	$\geqq$32/R	$\geqq$32/R	$\geqq$32/R	$\geqq$32/R	$\geqq$32/R	16/I
Amoxicillin/Clavulanic Acid	$\geqq$32/R	$\geqq$32/R	$\geqq$32/R	$\geqq$32/R	$\geqq$32/R	$\geqq$32/R	$\geqq$32/R	$\geqq$32/R	$\geqq$32/R	$\geqq$32/R	$\geqq$32/R	$\geqq$32/R	$\geqq$32/R	$\geqq$32/R	4/S
Piperacillin/Tazobactam	$\geqq$128/R	$\geqq$128/R	16/R	$\geqq$128/R	$\geqq$128/R	$\geqq$128/R	$\geqq$128/R	$\geqq$128/R	$\geqq$128/R	$\geqq$128/R	$\geqq$128/R	$\geqq$128/R	64/R	$\geqq$128/R	$\leqq$4/S
Cefazolin	$\geqq$64/R	$\geqq$64/R	$\geqq$64/R	$\geqq$64/R	$\geqq$64/R	$\geqq$64/R	$\geqq$64/R	$\geqq$64/R	$\geqq$64/R	$\geqq$64/R	$\geqq$64/R	$\geqq$64/R	$\geqq$64/R	$\geqq$64/R	$\leqq$4/S
Cefoxitin	$\geqq$64/R	$\geqq$64/R	$\geqq$64/R	$\geqq$64/R	$\geqq$64/R	$\geqq$64/R	$\geqq$64/R	$\geqq$64/R	$\geqq$64/R	$\geqq$64/R	$\geqq$64/R	32/R	32/R	32/R	8/S
Ceftriaxone	$\geqq$64/R	$\geqq$64/R	$\geqq$64/R	$\geqq$64/R	$\geqq$64/R	$\geqq$64/R	$\geqq$64/R	$\geqq$64/R	$\geqq$64/R	$\geqq$64/R	$\geqq$64/R	$\geqq$64/R	32/R	$\geqq$64/R	$\leqq$1/S
Cefepime	$\geqq$64/R	$\geqq$64/R	$\geqq$64/R	$\geqq$64/R	$\geqq$64/R	$\geqq$64/R	$\geqq$64/R	$\geqq$64/R	$\geqq$64/R	$\geqq$64/R	$\geqq$64/R	2/S	2/S	2/S	$\leqq$1/S
Aztreonam	$\geqq$64/R	$\geqq$64/R	$\geqq$64 R	$\geqq$64/R	$\geqq$64/R	$\geqq$64/R	$\geqq$64/R	$\geqq$64/R	$\geqq$64/R	$\geqq$64/R	$\geqq$64/R	$\geqq$64/R	$\geqq$64/R	$\geqq$64/R	$\leqq$1/S
Ertapenem	64/R	64/R	8/R	64/R	64/R	128/R	32/R	64/R	64/R	64/R	32/R	16/R	32/R	4/R	0.25/S
Imipenem	8/R	16/R	4/R	16/R	64/R	64/R	8/R	8/R	4/R	8/R	32/R	8/R	8/R	8/R	0.5/S
meropenem	32/R	64/R	64/R	64/R	8/R	16/R	8/R	32/R	16/R	128/R	64/R	8/R	8/R	8/R	0.25/R
Amikacin	$\geqq$64/R	$\leqq$2/S	$\geqq$64/R	$\leqq$2/S	$\geqq$64/R	$\geqq$64/R	$\geqq$64/R	$\leqq$2/S	$\leqq$2/S	$\leqq$2/S	$\geqq$64/R	4/S	$\leqq$2/S	4/S	$\leqq$2/S
Gentamicin	$\geqq$16/R	$\leqq$16/R	$\geqq$16/R	$\geqq$16/R	$\geqq$16/R	$\geqq$16/R	$\geqq$16/R	$\leqq$1/S	$\leqq$1/S	$\leqq$1/S	$\geqq$16/R	$\leqq$1/S	$\leqq$1/S	$\leqq$1/S	$\leqq$1/S
Tobramycin	$\geqq$16/R	$\geqq$16 R	$\geqq$16/R	$\geqq$16/R	$\geqq$16/R	$\geqq$16/R	$\geqq$16/R	$\leqq$1/S	$\leqq$1/S	$\geqq$1/S	$\geqq$16/R	$\leqq$1/S	$\leqq$1/S	$\leqq$1/S	$\leqq$1/S
Ciprofloxacin	$\geqq$4/R	1/R	$\geqq$4/R	1/R	$\geqq$4/R	$\geqq$4/R	0.5/I	$\geqq$4/R	$\geqq$4/R	$\geqq$4/R	$\geqq$4/R	$\leqq$0.25/S	$\leqq$0.25/S	$\leqq$0.25/S	$\leqq$0.25/S
Levofloxacin	$\geqq$8/R	1/I	$\geqq$8/R	1/I	$\geqq$8/R	$\geqq$8/R	4/R	$\geqq$8/R	$\geqq$8/R	$\geqq$8/R	$\geqq$8/R	0.5/S	0.5/S	0.5/S	0.5/S
Tigecycline	16/R	8/R	4/I	8/R	8/R	8/R	8/R	16/R	8/R	8/R	8/R	0.125/S	0.125/S	0.125/S	0.125/S
Nitrofurantoin	$\geqq$512/R	256/R	$\geqq$512/R	256/R	$\geqq$512/R	64/I	128/R	$\geqq$512/R	256/R	$\geqq$512/R	64/I	$\geqq$16/S	$\leqq$16/S	$\leqq$16/S	$\leqq$16/S
Trimethoprim/Sulfamethoxazole	$\geqq$320/R	$\geqq$320/R	$\geqq$320/R	$\geqq$320/R	$\leqq$20/S	$\geqq$320/R	$\geqq$320/R	$\geqq$320/R	$\geqq$320/R	$\geqq$320/R	$\geqq$320/R	$\geqq$20/S	$\leqq$20/S	$\leqq$20/S	$\leqq$20/S
Colistin	2/I	1/I	2/I	1/I	1/I	1/I	1/I	1/I	1/I	1/I	64/R	1/I	1/I	1/I	0.25/I

### MLST, virulence genes and plasmid replicon types

**Fig. 1:**
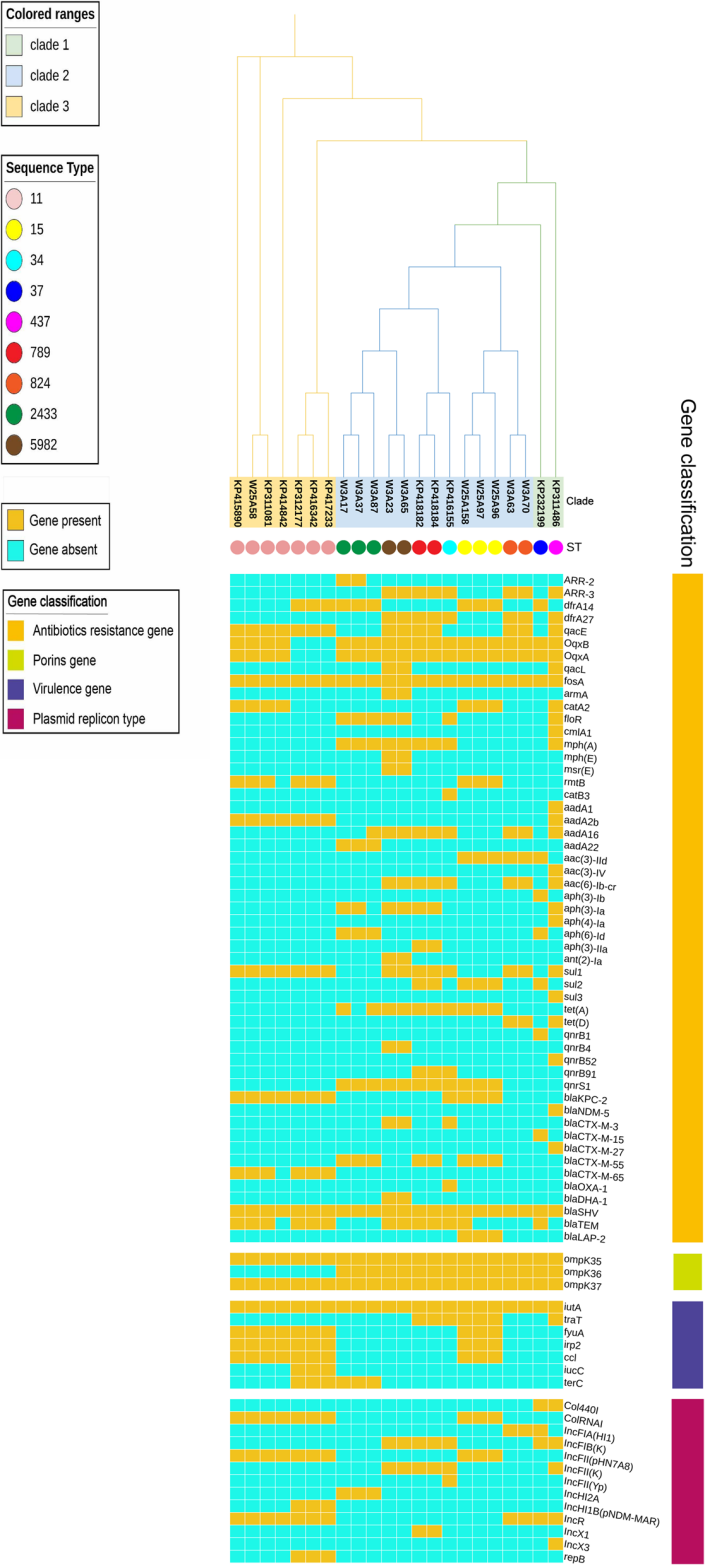
The Phylogenetic tree and gene prediction results of sequence types, virulence factors, plasmid replicon types, and antibiotics resistance genes in 22 CRKP genome. Heat map of 22 CRKP genomes showed the presence and absence of 53 antibiotic resistance genes, seven virulence factors and 13 plasmid types. Genomes of CRKP are represented on the X axis and gene names are listed on the Y axis. Yellow-colored squares indicate the presence of genes, and blue-colored squares indicate absence of genes

Figure 1 showed the general genome features of CRKP from HWW and clinical CRKP sequenced genomes in this study. Genetic analysis demonstrated that 11 CRKP from HWW belonged to five sequence types (STs), including ST11, ST15, ST824, ST2433 and ST3184. 11 clinical CRKP belonged to five sequence types, including ST11, ST34, ST37, ST437and ST789.

The distributions of virulence genes (VFs) among all CRKP from HWW were shown in Fig. 1. At least one VF was detected in each isolate. Among them, *iutA* (11/11, 100%) was the most prevalent VF in this study, following by *ccI* (4/11, 36.36%)*, fyuA* (4/11, 36.36%), *irp*2 (4/11, 36.36%), *terC* (3/11, 27.27%) and *traT* (3/11, 27.27%). Additionally, *iucC* was only detected in clinical CRKP isolates.

Overall, we found that all CRKP from HWW contained at least one plasmid replicon, covering seven different known plasmid incompatibility types in this study. F-type plasmid was highly prevalent across CRKP from HWW, including IncFIA, IncFIB and IncFII. In addition, Col440I, IncFII(Yp), IncHI1B(pNDM-MAR), IncX1, IncX3 and repB were only identified in clinical CRKP, and IncHI2A was only detected in CRKP from HWW, respectively.

### Determination of resistance genes and porin-associated genes

The distributions of antimicrobial resistance genes (ARGs) differed among CRKP from HWW (Fig. 1). Some ARGs were identified more than 70% among CRKP from HWW isolates, such as *fosA* (fosfomycin resistance gene, 100%), *tetA* (tetracycline resistance gene, 100%), *oqxA* and *oqxB* (fluoroquinolone resistance genes, 100%).

Only four CRKP from HWW isolates (W25A96, W25A97, W25A158 and W25A58) were detected to carry carbapenems resistance gene, *bla*_KPC-2_. The results of carbapenems resistance gene were verified by PCR. Other seven strains carried at least one non-carbapenemase β-lactamase gene targeted for detection in this study. Resistance genes found among the seven isolates without carbapenemase resistance gene were *bla*_DHA_, *bla*_TEM_, *bl*a_SHV_, *bla*_CTX-M_, *bla*_LAP_ and *bla*_OXA_ genes (Table 2). In total, deletion of porin-associated genes was detected in 100% of the CRKP from HWW isolates without carbapenemase resistance gene (Fig. 1 and Tables 2).

**Table 2:** Detection of resistance genes and porin-associated genes of 11 CRKP from HWW

Carbapenemase Resistance Gene^a^	Non-Carbapenemase *β*-Lactamase Gene	Porin Loss (ompK)^b^	Number of Strains (n)	MIC of Carbapenem
*bla*_KPC-2_	*bla*_CTX-M_, *bla*_SHV_ and *bla*_TEM_	36	1	ETP = 32 mg/L, IPM = 32 mg/L, MEM = 64 mg/L
	*bla*_CTX-M_, *bla*_SHV_ and *bla*_LAP_	/	1	ETP = 128 mg/L, IPM = 64 mg/L, MEM = 16 mg/L
	*bla*_CTX-M_, *bla*_SHV_ and *bla*_LAP_	/	1	ETP = 32 mg/L, IPM = 8 mg/L, MEM = 8 mg/L
	*bla*_CTX-M_, *bla*_SHV_, *bla*_TEM_ and *bla*_LAP_	/	1	ETP = 64 mg/L, IPM = 64 mg/L, MEM = 8 mg/L
-	*bla*_SHV_	/	2	ETP = 64 mg/L, IPM = 16 mg/L, MEM = 64 mg/L
	*bla*_CTX-M_ and *bla*_SHV_	/	1	ETP = 64 mg/L, IPM = 8 mg/L, MEM = 32 mg/L
	*bla*_CTX-M_ and *bla*_SHV_		1	ETP = 64 mg/L, IPM = 4 mg/L, MEM = 16 mg/L
	*bla*_CTX-M_ and *bla*_SHV_	/	1	ETP = 64 mg/L, IPM = 8 mg/L, MEM = 128 mg/L
	*bla*_CTX-M_, *bla*_SHV_, *bla*_DHA_ and *bla*_TEM_	/	1	ETP = 64 mg/L, IPM = 8 mg/L, MEM = 32 mg/L
	*bla*_CTX-M_, *bla*_SHV_, *bla*_DHA_ and *bla*_TEM_	/	1	ETP = 8 mg/L, IPM = 4 mg/L, MEM = 64 mg/L

MIC, Minimum Inhibitory Concentration; ETP, Ertapenem; IMP, Imipenem; MEM, Meropenem

^a^The symbol “-” denotes the gene was absent

^b^The symbol “/” denotes not applicable

Notably, we found two types of carbapenems resistance gene (seven isolates carrying *bla*_KPC-2_ and one isolates carrying *bla*_NDM-5_) in clinical CRKP isolates (8/11, 72.7%), which was a significant difference between CRKP from HWW and clinical settings (*p* < 0.05).

### Phylogenetic analysis

A total of 22 recoverable CRKP isolates were whole genome sequenced. The core-genome alignments were applied for phylogenetic tree reconstruction using maximum likelihood estimation (Fig. 1). All the 22 CRKP isolates were clustered into the three distinct phylogenetic clades (clade 1 to 3) in this study. Notably, clade 2 and clade 3 were mixtures of samples collected from both HWW and clinical settings. Among those clades, clade 1 included two isolates clinical CRKP isolates (belonged to ST37 and ST437), clade 2 included 10 CRKP isolates from HWW and three clinical CRKP isolates (belonged to ST15, ST34, ST789, ST824, ST2433 and ST3184), and clade 3 included six clinical CRKP isolates and one CRKP isolate from HWW (all isolates belonged to ST11). Furthermore, same sequence type was clustered in a subbranch relatively in clade 2.

### Conjugation experiments and plasmid replicon type analysis

In this study, three of four *bla*_KPC-2_-positive isolates (W25A96, W25A97 and W25A158) could transfer *bla*_KPC-2_ into EC600 successfully. Among them, the conjugation frequency of W25A96 was 1.58 × 10^–4^, W25A97 was 1.99 × 10^–4^, and W25A158 was 4.62 × 10^–4^. The presence of *bla*_KPC-2_ and plasmid replicon types was confirmed by PCR. Only IncFII pHN7A8 was found in those transconjugants. The results of antimicrobial susceptibility testing among recipient cell and transconjugants were shown in Table 1.

### Genetic Environment of the *bla*_KPC-2_ Gene

Horizontal gene transfer has been a major reason for rapid evolution in the resistance of bacteria, which leads to the capture of resistance genes [10]. The rapid spread of *bla*_KPC-2_ between different bacteria is due to the propagation of the *bla*_KPC-2_-positive plasmid with transposons and sequence elements (IS).

**Fig. 2:**
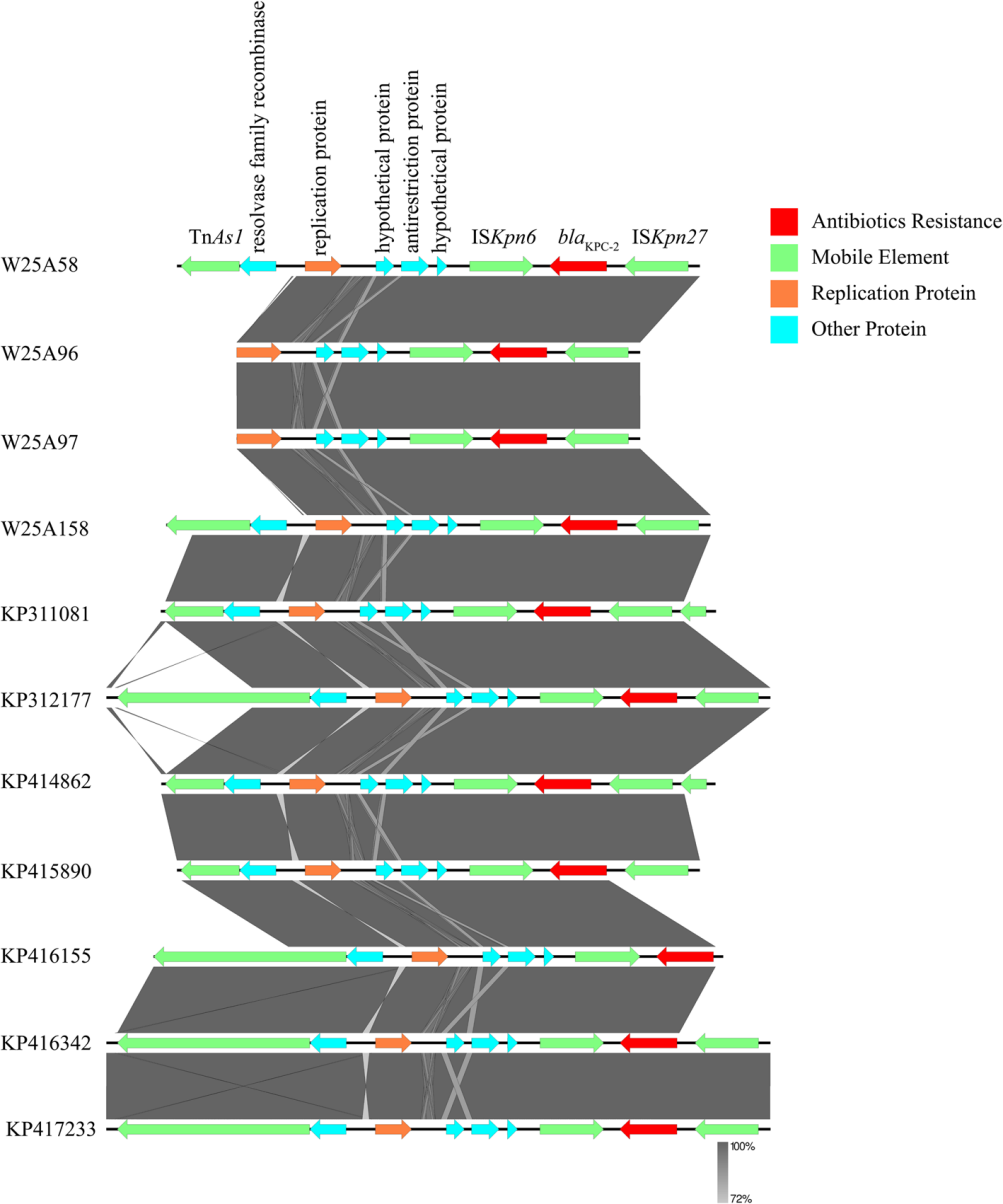
Genetic environments of *bla*_KPC-2._ Antibiotics resistance gene, mobile element gene, replication protein and other protein are colored in red, green, orange and blue, respectively

Here, we further detected the genetic environment of *bla*_KPC-2_. Four *bla*_KPC-2_-positive CRKP isolates from HWW shared same core structure with IS*Kpn27*-*bla*_KPC−2_-IS*Kpn6* in this study (Fig. 2). A IS*Kpn27* element was located in upstream of *bla*_KPC-2_. Furthermore, a IS*Kpn6* element was located downstream of *bla*_KPC-2_, followed by a hypothetical protein, an antirestriction protein, a hypothetical protein and a replication protein. Furthermore, the genetic environments of *bla*_KPC-2_ of CRKP isolates from HWW showed highly similar structure compared to *bla*_KPC-2_-positive clinical CRKP strains.

### Survivability-associated phenotypic characteristics

#### Serum resistance assay

**Fig. 3:**
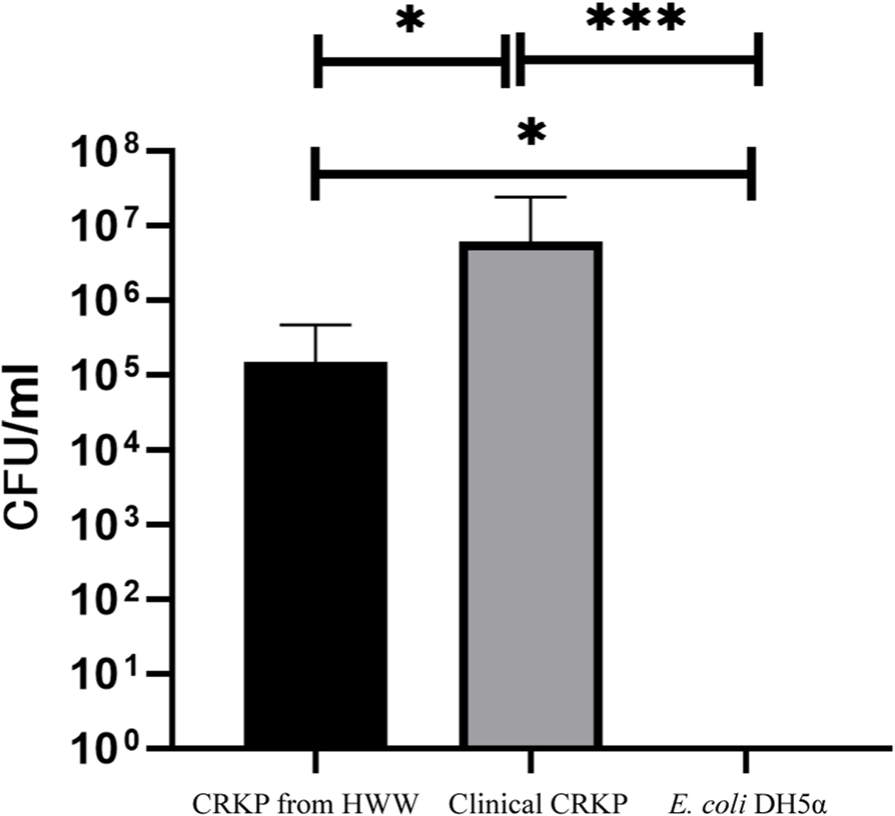
Resistance to serum bactericidal activity against 50% human serum. A total of 11 CRKP from HWW and 11 clinical CRKP strains were used for comparison. Serum resistance assay was performed by a 3-h incubation of CRKP and 50% human serum. *E. coli* DH5α was used as negative control. Unpaired two-tailed Student’s t-tests and Wilcoxon sign rank test were performed to analyze statistical significance. The error bars represent standard deviation (SD). Notes: *p < 0.05 and ***p < 0.0001

The results of serum resistance assay were shown in Fig. 3. All CRKP were tested in 50% human serum to investigate their capacity to resist the serum bactericidal activity. CRKP from HWW and clinical CRKP isolates showed significant growth compared to negative control, *E. coli* DH5α (*p* < 0.05). Group analysis revealed that clinical CRKP isolates had a stronger survivability compared to CRKP from HWW isolates (*p* < 005) in our study.

#### Survival assay

**Fig. 4:**
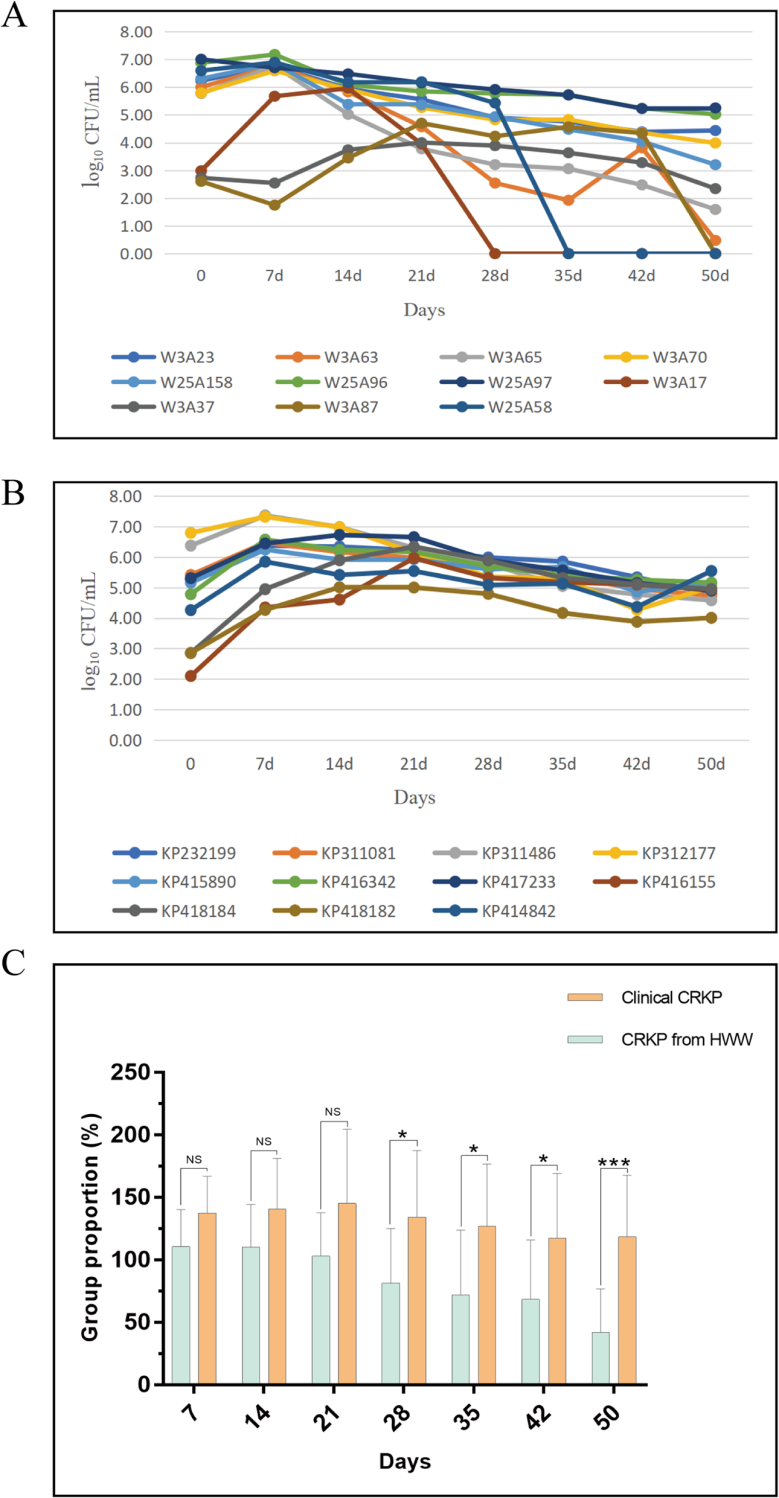
Survival assay of CRKP from HWW and clinical CRKP in the HWW during 50 days. (**A**) Growth trend of 11 CRKP from HWW. **B** Growth trend of 11 clinical CRKP. **C** Group analysis of growth proportion of every strain (compared to 0 day) between CRKP from HWW and clinical CRKP. 1 mL overnight bacterial culture (in LB broth) was suspended in duplicate in test tubes containing 40 mL of the autoclaved HWWs with shaking at 180 rpm. The number of bacteria was determined every 7 days in duplicate. Unpaired two-tailed Student’s t-tests and Wilcoxon sign rank test were performed to analyze the growth proportion of every strain (compared to 0 day) between CRKP from HWW and clinical CRKP. The error bars represent standard deviation (SD). Notes: NS: no significance, *: *p* < 0.05 and ***: *p* < 0.0001

The results of survival assay were presented in Fig. 4. 72.7% (8/11) of CRKP from HWW and all clinical CRKP survived in HWW for 50 days of monitoring. 81.8% 9/11) of CRKP from HWW and all clinical CRKP had an increase within seven days of incubation. 72.7% (8/11) of CRKP from HWW and 72.7% (8/11) of clinical CRKP showed a slight decrease after 14 days. After 50 days of incubation, 54.5% (6/11) of CRKP from HWW and all of clinical CRKP remained survive more than 50% of their original colony forming units. Group analysis revealed that CRKP from HWW had no significant difference in survival capability in autoclaved HWW compared to clinical CRKP (*p* > 005) within 21 days in our study. Clinical CRKP showed significant difference in survival capability in 28 days, 35 days, 42 days and 50 days compared to CRKP from HWW (*p* < 0.05, Fig. 4).

## Discussion

CRKP posed a severe clinical problem given the lack of therapeutic options available [2]. Nowadays, CRKP was detected in many environments, such as clinical settings, hospital wastewater, municipal wastewater and so on [5]. HWW was recognized as a putative reservoir for CRKP due to its selective pressure. Nowadays, there were many reports of CRKP from HWW around the world, such as Germany [11], United States [12], China [13], Romania [14], South Africa [6] and so on. Those findings indicated their possible spread in the kinds of water environments and the potential risk of human, livestock and wildlife colonization and/or infection associated with exposure to contaminated water sources. However, there was little research on the genomic diversification and functional characteristics of CRKP from HWW, especially in China [6]. Here, we reported the whole genome sequences and comparative functional analysis of CRKP from HWW in a Chinese teaching hospital. These genomes represent a significant addition of genomic data from the genus and could serve as a valuable resource for future genomic studies about CRKP from HWW.

In the present study, 1.39 × 10^4^ CFU/ml of CRKP were detected from HWW approximately and a total of 11 strains were recovered. The detection rate was higher than that in South Africa and lower than that in urban wastewater in eastern India [6, 15]. Antibiotic selection pressure could affect the fraction of bacterial genera potentially [16]. So, this discrepancy might be related to the different antibiotic selection pressure in different regions, which deserved to investigate further.

Molecular typing is usually used to identify clonal relationships among bacteria and applied to detect the population structure of CRKP strains with respect to their specificity for infecting humans or animals [17]. Previous studies reported that ST11-CRKP was disseminated widely in clinical settings across China [18, 19]. Meanwhile, ST11 was identified as common STs in this hospital (Fig. 1) and Fujian province as described previously [20, 21]. In this study, our results revealed that CRKP from HWW possessed high diversity at the level of sequence types (Fig. 1). As similar to previous studies, there is diversity of ST types of CRKP from HWW and their distribution and dominant groups are different from those in the clinical environment [6, 22]. Interestingly, our findings of comparative genomic analysis showed that clinical CRKP and CRKP from HWW have an intersection in same clade (clade 2 and clade 3) and the *bla*_KPC-2_ were all found in those STs (Fig. 1). Those similar circulating STs might suggest the dissemination of this sequence type from hospital settings to aquatic environments and the possibility of spread clonally of *bla*_KPC-2_-carrying CRKP strains. Similar to previous report, the ST of non-carbapenemase-producing CRKP (NC-CRKP) also showed diversity, which distinguished from the dominant STs of CRKP carrying carbapenemase genes [23]. In addition, the phylogenetic tree showed that CRKP from HWW possessed high diversity at the level of phylogeny that associated with sequence types (Fig. 1), which was similar to previous study [5]. Combined with the results of profiles of resistance genes, virulence genes and plasmid replicon types, these findings suggested that the most CRKP from HWW and clinical CRKP share variant genetic backgrounds with different STs and might belong to the distance ancestral lineage(s).

Carbapenems resistance was mainly linked to the acquisition of carbapenems resistance genes (such as *bla*_KPC_, *bla*_NDM_, *bla*_VIM_), efflux pumps genes and mutations in outer membrane porins [24, 25]. In this study, CRKP from HWW exhibited a diversity of antibiotics resistance gene profiles, particularly including carbapenems resistance genes in four isolates (Fig. 1). But most of CRKP from HWW (7/11, 63.6%) in current study did not carry any carbapenems resistance genes (Fig. 1). This is an interesting phenomenon. NC-CRKP confers carbapenem resistance association with chromosomal mutations (such as porin gene mutation, and overproduction of efflux pump) and acquired non-carbapenemase resistance mechanisms (acquisition or upregulation of a β-lactamase such as ESBL or AmpC β-lactamase) [26, 27]. Meanwhile, loss or deficiency of outer membrane porins were associated with carbapenem resistance [28]. In our study, some ESBLs genes (such as *bla*_SHV_), AmpC β-lactamase genes (such as *bla*_DHA-1_) and outer membrane porins genes could be detected in those NC-CRKP strains from HWW (Fig. 1 and Table 2). Therefore, future studies are needed to verify whether it’s a feature of CRKP in HWW by expanding the sample size and warranted to confirm the contribution of these factors to carbapenem resistance for NC-CRKP from HWW. [29]. Additionally, 90.9% CRKP from HWW had resistance to tigecycline, which was not consistent with the previous report [30]. Tigecycline resistance has inevitably emerged over the recent years, mostly identified among extensively drug-resistant and carbapenem-resistant isolates [31]. However, any gene associated with tigecycline resistance has not been found in our study. There may be other undiscovered mechanisms of tigecycline resistance, which are worth further exploration. Interestingly, the transconjugants carried *bla*_KPC-2_ were all sensitive to cefepime, which was similar to previous studies [32, 33]. The potential mechanism of cefepime-resistance change through conjugation is worth further study.

The dissemination of carbapenems resistance is associated with horizontal transfer of carbapenems resistance genes [34]. In this study, our study demonstrated that most of *bla*_KPC-2_-harboring plasmid in CRKP from HWW (3/4) was transferrable with high conjugation frequency. Given the high transferability of *bla*_KPC-2_-harboring-IncFII pHN7A8 plasmid in CRKP from HWW, the spread of *bla*_KPC-2_-harboring plasmids into other bacteria had the potential to cause the spread of carbapenems resistance in HWW. Meanwhile, many studies characterized the genetic environments of *bla*_KPC-2_ and various mobile genetic elements played a critical role in the rapid spread of *bla*_KPC-2_ [35, 36]. In this study, we found that the genetic environments of *bla*_KPC−2_ shared the core structure with IS*Kpn27*-*bla*_KPC−2_-IS*Kpn6*, which was similar to the previous study [37]. Bacteria could acquire preexisting resistance determinants to promote drug resistance, which was achieved through the concerted activities of mobile genetic elements (such as IS, transposons) and plasmids and integrative conjugative elements [38]. The main genetic structure containing transposons and IS could enhance the spread of the *bla*_KPC_-type genes in different plasmid scaffolds [39]. Thus, our results indicated that these mobile elements played a key role in the dissemination of *bla*_KPC−2_ in CRKP from HWW. Current study indicated the semblable genetic environments of *bla*_KPC-2_ between CRKP from HWW and clinical settings, and our previous study also found similar plasmids carrying *bla*_KPC−2_ in this hospital in clinical CRKP [40], which was also evidence that the possibility of the dissemination of CRKP from hospital settings to aquatic environments.

To gain understanding of CRKP from HWW survivability, the 11 CRKP from HWW along with 11 clinical CRKP were assessed for their survivability to serum and hospital wastewater in vitro. CRKP from HWW had a weaker survivability in serum and similar survivability in HWW compared to clinical CRKP in this study (Fig. 3 and Fig. 4). Serum resistance was one of the major survival mechanisms of bacteria enabling them to survive in the bloodstream of the host [41]. Our study indicated that clinical CRKP might more potentially contribute to the bloodstream infections than CRKP from HWW. Additionally, previous study suggested some abiotic and biotic ecological factors were shown to determine CRKP survival and multiplication when CRKP isolates came in the environment [42]. Our results indicated that CRKP from HWW and clinical CRKP might have the potential to persist in HWW for a long time and CRKP from HWW was a potential risk of carbapenems resistance dissemination. Meanwhile, more research to identify mitigating measures is needed to effectively track the dissemination of these bacteria from different environmental resistomes to humans.

There was a limitation to our study. The CRKP from HWW were isolated from a single hospital with small sample size. Further study needs to increase the sample size, including new CRKP from HWW from other hospital in this area and other provinces in China to make the results of comparative genomics analysis more representatives in the future.

## Conclusions

In this study, we analyzed the molecular characteristic of CRKP isolates from HWW in a Chinese teaching hospital using whole genome sequencing. High diversity at the level of sequence types and phylogeny were found. Only one type of carbapenems resistance genes – *bla*_KPC-2_ was detected and most of them located on a transferrable plasmid with high conjugation frequency. All of *bla*_KPC−2_ in this study shared same genetic environment’s structure. Moreover, in our study, CRKP from HWW showed lower survivability in serum and similar survivability in hospital wastewater compared to clinical CRKP. These findings could add to the data on the genomic and functional characterization of CRKP from HWW in China and provide a good pilotage for further in-depth studies of CRKP’s from HWW properties and careful surveillance of its emergence across different ecologies.

## Methods

### Bacterial isolates

The hospital wastewater was collected from the raw sewage influent of on-site hospital wastewater treatment from Fujian medical university union hospital (Fujian province, China), and the collection points were only at locations specific to this hospital [43]. Briefly, a total of three hospital wastewater samples were taken between November 3 and November 25, each ten days apart. The samples were collected and then transported to laboratory on ice for immediate processing during half-hour. After rinsing bottles twice with target samples, 1 L wastewater samples were filled into sterile plastic bottles. After being thoroughly mixed, wastewater samples were serially diluted. Then 1 mL serial diluted wastewater samples were plated on Luria–Bertani (LB) agar (Sangon Biotech (Shanghai) Co., Ltd, China) (supplemented with Imipenem at 4 µg/mL) to select carbapenem resistance bacteria followed by incubation at 37 ℃ for 24 h. Wastewater samples of each dilution were plated on LB agar in triplicates.

The recovered bacterial species were identified using MALDI-TOF (Bruker Daltonics, Germany). Briefly, every single clone was grown on Columbia blood agar (Autobio, China) and incubated for 18 h at a temperature of 36 °C. Then, fresh bacterial colonies were coated on the MALDI target plates (Bruker Daltonik, Germany) and covered with 1 μL chemical matrix (α-cyano-4-hydroxycinnamic acid). Collection of spectra (2–20 kDa) was performed in linear and positive mode of each point by FlexControl software (Bruker Daltonik). If a colony was unidentified, 16S rRNA sequencing was performed [20]. The primers of 16S rRNA were listed in Table S3 (Supplementary Material). The strains identified to *Klebsiella pneumoniae* using MALDI-TOF and 16 s rRNA sequencing were collected and tested for carbapenem susceptibility performed by broth dilution method. The isolate, whose MIC value of imipenem was 4 μg/mL or higher, was recorded as a positive confirmation result for CRKP*.*

For comparative analysis, 11 clinical CRKP isolates were collected from Fujian Medical University Union Hospital between November to December, 2019. The clinical CRKP strains were then randomly assigned in a ratio into matched arm using random number generators (SPSS, version.20) to avoid the selection bias. The microbiological characteristics and antibiotics profiles of 11 clinical CRKP isolates were listed in Table S1 and Table S2 (Supplementary Material).

### Antimicrobial susceptibility testing

The antimicrobial susceptibility testing of the CRKP from HWW, recipient and transconjugants was performed by Vitek-2 (Vitek-AST-GN16) (BioMerieux, France) according to the Clinical and Laboratory Standards Institute guidelines (CLSI, 2022). The antibiotics included ampicillin, amoxicillin/clavulanic acid, piperacillin/tazobactam, cefazolin, cefoxitin, ceftriaxone, cefepime, aztreonam, amikacin, gentamicin, tobramycin, ciprofloxacin, levofloxacin, nitrofurantoin, and trimethoprim/sulfamethoxazole. Among them, MICs of ertapenem (Macklin, China), imipenem (Solarbio, China), meropenem (Solarbio, China), tigecycline (Macklin, China) and colistin (Solarbio, China) were determined using broth dilution method. *E. coli* ATCC 25,922 was used as quality control.

### Whole genome sequencing and analysis

The genomic DNA samples were extracted from the CRKP from HWW and clinical CRKP isolates by TIANamp Bacteria DNA Kit (Tiangen, Beijing), sequenced using the Illumina NovaSeq platform in Shanghai Personal Biotechnology Co., Ltd (Shanghai, China), with a paired-end mode of 2 × 150 bp. Raw data was checked for quality with FastQC (version.0.11.7). AdapterRemoval (version.2.2.2) was used for trimming of adapter sequences and low-quality bases and SOAPec (version.2.03) was used for quality correction based on K-mer frequency before data assembly. The filtered raw data were assembled to contigs and scaffolds using SPAdes (version.3.12.0) and A5-MiSeq (version.20160825). The base correction was performed using Pilon (version.1.18). The depth of coverage was ranged from 224-fold for 10,459,852 reads to 292-fold for 10,256,762 reads and the average depth of coverage was 260-fold.

Gene content analysis was performed using tools available through the Center for Genomic Epidemiology (https://cge.cbs.dtu.dk/services/). Specifically, acquired resistance genes and known chromosomal mutations conferring antibiotic resistance were identified using ResFinder [44]. Plasmid types were identified using PlasmidFinder [45]. Virulence genes were identified using VirulenceFinder [46]. The multilocus-sequence typing was identified on MLST (https://cge.food.dtu.dk/services/MLST/).

The genetic environment of *bla*_KPC-2_ was predicted in RAST tool (https://rast.nmpdr.org/rast.cgi). Comparative analysis of the genetic environment of *bla*_KPC-2_ in CRKP from HWW and clinical CRKP was performed using Easyfig (version.2.1).

### Detection of carbapenemase genes

To verify the prediction results of carbapenemase genes using whole genome sequencing, the presence of carbapenemase genes was identified by PCR as described previously [20], including *bla*_KPC_, *bla*_NDM_, *bla*_VIM_, *bla*_IMP_, *bla*_OXA-48_ and *bla*_GES_. The primers of carbapenemase genes are listed in Table S3 (Supplementary Material).

### Phylogenetic analysis

Core genomes were defined as described previously [47]. Based on the comparative analysis of 11 CRKP from HWW and 11 clinical CRKP isolates, core genomes were selected as single-copy core-genome for sequence alignment using mafft (version.7.429) [48]. The set of single-copy core-genome clusters detected was entered into the pipeline to identify high-quality phylogenetic markers and infer a core genome phylogeny through a maximum-likelihood tree search. Maximum likelihood phylogeny of core of 22 CRKP isolates were inferred from the alignment using FastTree (version.2.1.11) with Whelan-and-Goldman 2001 model and 1000 bootstrap replicates, respectively [49]. The resulting phylogenetic tree was visualized using iTOL.

### Conjugation experiments and plasmid replicon types analysis

Conjugation experiments were performed to test the transferability of *bla*_KPC-2_-harboring plasmids using filter mating [50]. Rifampin-resistant *E. coli* EC600 (stored in our laboratory) was used as the recipient. The mating mixture was washed from the filter and spread onto MH agar (Sangon Biotech (Shanghai) Co., Ltd, China) containing sodium rifampicin at 600 μg/mL and imipenem at 1 μg/mL. The transconjugants harbored *bla*_KPC-2_ were selected and identified by PCR [20]. *bla*_KPC-2_ was amplified using primers listed in Table S3 (Supplementary Material). The conjugation frequency (CF) was calculated as follow [50]:

$$\mathrm{CF}=\frac{\mathrm{Number\ transconjugants}(\mathrm{CFU}/\mathrm{mL})}{\mathrm{Numbers\ of\ donor\ and\ recipient\ cells}(\mathrm{CFU}/\mathrm{mL})}$$

The transconjugants harbored *bla*_KPC-2_ were examined for the presence of three plasmid replicons, combining the results of gene prediction, using the PCR as mentioned above [20]. Three plasmid replicons were amplified using primers listed in Table S3 (Supplementary Material).

### Survivability-associated phenotypic characteristics

#### Serum resistance assay

Serum resistance assay of 11 CRKP from HWW and 11 clinical CRKP was tested using 50% human serum (Precision BioMedicals Co., Ltd, China) for a 3-h incubation as previous studies [41, 51]. Briefly, 5 μL of an overnight bacterial culture was added to 495 μL of LB broth and centrifuged at 9000 rpm for 3 min. Then the pellet obtained was re-suspended in 1 × phosphate-buffered saline. 30 μL of the above bacterial inoculum and 270 μL of the 50% human serum were added and mixed in a 96-well plate for a 3-h incobation. Then the mixture was taken for serial dilutions, plated on LB agar and cultured overnight. The growth in the serum of strain was detected as the total number of CFU/mL recovered per well [41]. The serum resistance assay was performed twice in triplicates. *E. coli* EC505 (stored in our laboratory) was used as the positive control and *E. coli* DH5α was used as the negative control [41, 52].

#### Survival assay

The survival assay of CRKP from HWW and clinical CRKP isolates was monitored for 50 days at room temperature (22 ± 2 ℃). HWW was filtered using membrane filter with 0.25 µm size. HWW was partitioned per 200 mL into bottles and autoclaved (121 °C for 20 min). 1 mL overnight bacterial culture (in LB broth) was suspended in duplicate in test tubes containing 40 mL of the autoclaved HWWs. Tubes were rotated at 180 rpm using a StuartTube Rotator THZ-100 (YiHeng, Shanghai, China). The number of bacteria was determined on LB agar in duplicate, which incubated at 35 ℃ for 24 h at 1-week intervals, and bacterial concentration was expressed as log_10_ CFU/mL [53]. For group comparison, the number of every strain was counted by growth proportion in 7, 14, 21, 28, 35, 42 and 50 days compared to the original colony forming units (0 day) [53].

#### Statistical analysis

Graphpad prism v.7 was used for statistical analysis. Unpaired two-tailed Student’s t-tests and Wilcoxon sign rank test (unequal variances) were performed to analyze statistical significance for serum resistance assay and survival assay. Only *p* < 0.05 was considered statistically significant.

## Supplementary Information

**Additional file 1:** **Table S1.**Main microbiological characteristics of 11 clinical CRKP isolates. **Table S2.** AntimicrobialSusceptibility Testing of 11 clinical CRKP isolates. **Table S3.** Nucleotide sequences ofprimers used in this study.

## Data Availability

The genome sequences of 11 CRKP from HWW and 11 clinical CRKP were deposited in GenBank under BioProject PRJNA854359 (https://www.ncbi.nlm.nih.gov/bioproject/PRJNA854359).

## Abbreviations

CRKP: Carbapenem-resistant *Klebsiella pneumoniae*
HWW: Hospital wastewater
ESBL: Extended spectrum beta-lactamase
MLST: Multilocus sequence typing
ST: Sequence type
MIC: Minimum inhibitory concentration
