# Supplementary material for: Genomic and functional characterization of carbapenem-resistant Klebsiella pneumoniae from hospital wastewater
Source: BMC Microbiol. 2023 Apr 24;23:115. doi: 10.1186/s12866-023-02862-5 (PMC10124015; doi:10.1186/s12866-023-02862-5)
Supplement: Supplementary file 1 — Additional file 1: Table S1.Main microbiological characteristics of 11 clinical CRKP isolates. Table S2. AntimicrobialSusceptibility Testing of 11 clinical CRKP isolates. Table S3. Nucleotide sequences ofprimers used in this study. [file 12866_2023_2862_MOESM1_ESM.doc]

Genomic and Functional Characterization of Carbapenem-resistant *Klebsiella pneumoniae* From Hospital Wastewater

Zhiqiang Xie^1,#^, Jiangqing Huang^1,#^, Shengcen zhang^1^, BinBin Xu^1^, Qianwen Zhang^1^, Bin Li^1,*^

1 Department of Clinical Laboratory, Fujian Medical University Union Hospital, Fuzhou, Fujian, 350001, China

# ZhiqiangXie and Jiangqing Huang contribute equally to the article.

*Correspondence

Prof. Bin Li

Department of Clinical Laboratory, Fujian Medical University Union Hospital, 29 Xinquan Rd., Fuzhou, Fujian, 350001. China

E-mail: leonlee307@hotmail.com

Table

Table S1. Main microbiological characteristics of 11 clinical CRKP isolates

| Isolate | Specimen | Word | Collection data |
| --- | --- | --- | --- |
| KP232199 | [Faeces](../../../../D:%5CDict%5C9.1.2.0%5Cresultui%5Chtml%5Cindex.html%23%5Cjavascript:%3B) | [Department of hematology](../../../../D:%5CDict%5C9.1.2.0%5Cresultui%5Chtml%5Cindex.html%23%5Cjavascript:%3B) | 2020.11 |
| KP311081 | Blood | Intensive Care Unit | 2020.11 |
| KP311486 | Blood | Intensive Care Unit | 2020.11 |
| KP312177 | Blood | Intensive Care Unit | 2020.11 |
| KP414842 | Blood | Intensive Care Unit | 2020.11 |
| KP415890 | Blood | Intensive Care Unit | 2020.11 |
| KP416155 | Blood | Department of cardiac surgery | 2020.11 |
| KP416342 | Blood | Intensive Care Unit | 2020.11 |
| KP417233 | Blood | Intensive Care Unit | 2020.12 |
| KP418184 | Blood | [Department of hematology](../../../../D:%5CDict%5C9.1.2.0%5Cresultui%5Chtml%5Cindex.html%23%5Cjavascript:%3B) | 2020.12 |
| KP418182 | Blood | [Department of hematology](../../../../D:%5CDict%5C9.1.2.0%5Cresultui%5Chtml%5Cindex.html%23%5Cjavascript:%3B) | 2020.11 |

Table S2. Antimicrobial Susceptibility Testing of 11 clinical CRKP isolates

| Antibiotics  (MICs(mg/L)antimicrobial susceptibility | Aantimicrobial Susceptibility Testing of clinical CRKP isolates | | | | | | | | | | |
| --- | --- | --- | --- | --- | --- | --- | --- | --- | --- | --- | --- |
|  |  |  |  |  |  |  |  |  |  |  |  |
|  | KP232199 | KP311081 | KP311486 | KP312177 | KP415890 | KP416342 | KP417233 | KP416155 | KP418184 | KP418182 | KP414842 |
| Ampicillin | ≧32/R | ≧32/R | ≧32/R | ≧32/R | ≧32/R | ≧32/R | ≧32/R | ≧32/R | ≧32/R | ≧32/R | ≧32/R |
| Amoxicillin/Clavulanic Acid | ≧32/R | ≧32/R | ≧32/R | ≧32/R | ≧32/R | ≧32/R | ≧32/R | ≧32/R | 16/I | 16/I | ≧32/R |
| Piperacillin/Tazobactam | ≧128/R | ≧128/R | ≧128/R | ≧128/R | ≧128/R | ≧128/R | ≧128/R | ≧128/R | 64/I | 8/S | ≧128/R |
| Cefazolin | ≧64/R | ≧64/R | ≧64/R | ≧64/R | ≧64/R | ≧64/R | ≧64/R | ≧64/R | ≧64/R | ≧64/R | ≧64/R |
| Cefoxitin | ≧64/R | ≧64/R | ≧64/R | ≧64/R | ≧64/R | ≧64/R | ≧64/R | ≧64/R | ≧64/R | ≧64/R | ≧64/R |
| Ceftriaxone | ≧64/R | ≧64/R | ≧64/R | ≧64/R | ≧64/R | ≧64/R | ≧64/R | ≧64/R | ≧64/R | ≧64/R | ≧64/R |
| Cefepime | ≧64/R | ≧64/R | ≧64/R | ≧64/R | ≧64/R | ≧64/R | ≧64/R | ≧64/R | ≧64/R | ≧64/R | ≧64/R |
| Aztreonam | ≧64/R | ≧64/R | 32/R | ≧64/R | ≧64/R | ≧64/R | ≧64/R | ≧64/R | ≧64/R | ≧64/R | ≧64/R |
| Ertapenem | 64/R | 128/R | 128/R | 128/R | 128/R | 128/R | 128/R | 32/R | 32/R | 8/R | 8/R |
| Imipenem | 8/R | 64/R | 128/R | 64/R | 64/R | 64/R | 64/R | 32/R | 2/I | 1/S | 64/R |
| Meropenem | 8/R | 8/R | 128/R | 8/R | 32/R | 8/R | 128/R | 64/R | 8/R | 0.5/S | 8/R |
| Amikacin | ≦2/S | ≧64/R | ≧16/R | ≧64/R | ≧64/R | ≧64/R | ≧64/R | ≦2/S | ≦2/S | ≦2/S | ≧64/R |
| Gentamicin | ≧64/R | ≧16/R | ≧16/R | ≧16/R | ≧16/R | ≧16/R | ≧16/R | ≦1/S | ≦1/S | ≦1/S | ≧16/R |
| Tobramycin | ≧16/R | ≧16/R | ≧4/R | ≧16/R | ≧16/R | ≧16/R | ≧16/R | 8/I | 8/I | 8/I | ≧16/R |
| Ciprofloxacin | 1/R | ≧4/R | ≧8/R | ≧4/R | ≧4/R | ≧4/R | ≧4/R | ≧4/R | ≧4/R | ≧4/R | ≧4/R |
| Levofloxacin | 1/I | ≧8/R | ≧8/R | ≧8/R | ≧8/R | ≧8/R | ≧8/R | 4/R | ≧8/R | ≧8/R | ≧8/R |
| Tigecycline | 2/S | 2/S | 4/I | 2/S | 2/S | 1/S | 0.25/S | 1/I | 8/R | 8/R | 4/I |
| Nitrofurantoin | 256/R | ≧512/R | ≧512/R | ≧512/R | ≧512/R | ≧512/R | ≧512/R | 128/R | ≧512/R | ≧512/R | ≧512/R |
| Trimethoprim/Sulfamethoxazole | ≧320/R | ≦20/S | ≧320/R | 80/R | ≦20/S | 80/R | 80/R | ≧320/R | ≧320/R | ≧320/R | ≦20/S |
| Colistin | 0.5/I | 0.5/I | 1/I | 0.5/I | 1/I | 1/I | 2/I | 1/I | 1/I | 1/I | 0.5/I |

Table S3. Nucleotide sequences of primers used in this study

| Primer name | Sequence (5' to 3' ) | Size of product (bp) | References |
| --- | --- | --- | --- |
| *bla*_KPC_-F | GCTACACCTAGCTCCACCTTC | 709 | 1 |
| *bla*_KPC_-R | GCATGGATTACCAACCACTGT |
| Col RNAⅠ-F | TTTCAAGAAGAGCCATCTCT | 130 | This Study |
| Col RNAⅠ-R | CATAAATATTTTTCTCCCTGGG |
| IncFⅡ (pHN7A8)-F | GAGAAATGCATCCCAGCAAC | 253 | This Study |
| IncFⅡ (pHN7A8)-R | GCTGAAATCTATAACCTTCGC |
| *bla*_NDM_-F | CACCTCATGTTTGAATTCGCC | 951 | This Study |
| *bla*_NDM_-R | CTCTGTCACATCGAAATCGC |
| *bla*_IMP_-F | TCACATTTCCATAGCGACAG | 450 | This Study |
| *bla*_IMP_-R | AGTGGTACTTTTTTTGCTTTCAT |
| *bla*_VIM_-F | GATGGTGTTTGGTCGCATA | 390 | This Study |
| *bla*_VIM_-R | CGAATGCGCAGCACCAG |
| *bla*_GES_-F | GTTTTGCAATGTGCTCAACG | 371 | This Study |
| *bla*_GES_-R | TGCCATAGCAATAGGCGTAG |
| *bla*_OXA-48_-F | TTGGTGGCATCGATTATCGG | 744 | This Study |
| *bla*_OXA-48_-R | GAGCACTTCTTTTGTGATGGC |
| 16s rRNA-27F | AGAGTTTGATCCTGGCTCAG | 1400 | 2 |
| 16s rRNA-1492R | GGTTACCTTGTTACGACTT |

Reference

1. Wolter DJ, Kurpiel PM, Woodford N, Palepou MF, Goering RV, Hanson ND. Phenotypic and enzymatic comparative analysis of the novel KPC variant KPC-5 and its evolutionary variants, KPC-2 and KPC-4. *Antimicrobial agents and chemotherapy.* 2009;53(2):557-562.

2. Lee MJ, Jang SJ, Li XM, et al. Comparison of rpoB gene sequencing, 16S rRNA gene sequencing, gyrB multiplex PCR, and the VITEK2 system for identification of Acinetobacter clinical isolates. *Diagnostic microbiology and infectious disease.* 2014;78(1):29-34.
